# Numerical Assessment Tool to Measure Realism in Clinical Simulation

**DOI:** 10.3390/ijerph20032247

**Published:** 2023-01-27

**Authors:** Gleyvis Coro-Montanet, María Jesús Pardo Monedero, Julia Sánchez Ituarte, Helena Wagner Porto Rocha, Carmen Gomar Sancho

**Affiliations:** 1 Preclinical Dentistry Department, School of Biomedical and Health Sciences, Universidad Europea, 28670 Madrid, Spain; 2 Health Sciences Facilities, Universidad Europea, 28670 Madrid, Spain; 3 Researcher SIMLAB Group, Universidad Europea, 28670 Madrid, Spain

**Keywords:** simulation-based learning, high-fidelity simulation, realism, fidelity

## Abstract

Realism is indispensable in clinical simulation learning, and the objective of this work is to present to the scientific community the methodology behind a novel numerical and digital tool to objectively measure realism in clinical simulation. Indicators measuring accuracy and naturality constitute ProRealSim v.1.0 (Universidad Europea, Madrid, Spain) which allows the assessing of attained realism for three dimensions: simulated participant, scenography, and simulator. Twelve experts in simulation-based learning (SBL) analyzed the conceptual relevance of 73 initial qualitative indicators that were then reduced to 53 final indicators after a screening study evaluating eight medical clinical simulation scenarios. Inter- and intra-observer concordance, correlation, and internal consistency were calculated, and an exploratory factorial analysis was conducted. Realism units were weighted based on variability and its mathematical contribution to global and dimensional realism. A statistical significance of *p* < 0.05 was applied and internal consistency was significant in all cases (raw_alpha ≥ 0.9698094). ProRealSim v.1.0 is integrated into a bilingual, free, and open access digital platform, and the intention is to foster a culture of interpretation of realism for its better study and didactic use.

## 1. Introduction

Simulation is a powerful educational resource that allows the training of health professionals in a risk-free context, benefiting students, care providers, and, ultimately, patients. This methodology allows the creation of fictional environments that replicate real situations and, the more realistic the situation presented and perceived, the better the immersion of the participants in the simulation and the attainment of learning and training objectives [1].

The realism of a scenario depends on the establishment of a close association between the simulated situation and the students’ perception of fidelity/realism. As stated by Nanji et al. (2013) and Mills et al. (2018), high levels of realism imply stronger commitment of learners [2,3]; however, realism and fidelity definitions convey some theoretical and practical vagueness [4].

In terms of assessing realism and fidelity, some authors [4,5] state that the research carried out in this field is based on subjective measurements and use small sample sizes with non-validated instruments.

The improvement of realism by introducing commercial or self-developed physical resources favor the performance of the student [3]. Alsaad et al. obtained significant results in realism and competencies development in trauma situations and crisis management [6] through its deployment. Other studies address the increase in realism using high-fidelity simulators that mimic tactile sensations, sounds, real physiological responses, and other feedback mechanisms [7]. Additionally, complementing the scenarios with audiovisual resources [8] may help improve realism in a way that the mannequin/simulator itself may not accomplish alone [9].

Current high-fidelity mannequins/simulators utilized in the fields of medicine and nursing allow the replication of physiological functions such as breathing and responding to medical and/or pharmacological interventions, in addition to mimicking physical signs such as bleeding or swelling during invasive procedures [10].

Additionally, scenic realism is considered one of the most important aspects in clinical simulation programs [11]. The faithful recreation of simulated environments using simulators and simulated participants through acting [12] improves learning transfer [13], cognitive retention [14], and student self-efficacy and confidence [15,16].

The absence of clear definitions of concepts related to realism and fidelity, combined with frail methodology in studies and evaluations on the topic, may eventually affect the design, evaluation, planning, and logistics of simulated practices. Furthermore, it may also prevent knowing the true impact of fidelity on learning in simulated practices [4].

The objective of this paper is to present the process that resulted in the development of ProRealSim v.1.0, a numerical assessment tool to measure attained realism in clinical simulation for three main dimensions: simulated participant, scenography, and simulator. Numeric indices assess the precision and naturality of several indicators that characterize the three main dimensions, supported by statistical scrutiny. By presenting it to the academic community that applies Simulation Based Learning (SBL), the intention is to set the groundwork and present the methodology behind the numerical approach, so it is validated and available for use and further refinement, ultimately contributing to the enhancement of the simulation methodology worldwide.

## 2. Materials and Methods

The research was carried out between 2020 and 2022 and funded by Universidad Europea—Investigation and Ethical Committee approval code 2020UEM39. The purpose was to conduct a screening study to detect relevant factors when assessing realism in clinical simulation, narrowing down possible indices, indicators, units, and dimensions.

### 2.1. Qualitative Study: Delphi Method

The theoretical construct anchoring ProRealSim v.1.0 started with the analysis of 73 indicators [17] developed by experts from the simulation center at Universidad Europea, based on the concept of Rudolph et al., 2014 [18], who proposed a disjunction between the terms fidelity and realism. All experts are engaged to a solid simulation program active for more than 5 years. These indicators touch on the realism/fidelity of three dimensions: the simulated participant, the scenography, and the simulator. These indicators originated from literature searches, systematic reviews, and consensus of focus groups on the subject. Afterward, a three-round Delphi study was conducted with the participation of 3 content reviewers and 12 experts from recognized Spanish simulation centers, namely the Universidad Europea, Universidad de Barcelona, Universidad de VIC—Manresa´s Simulation Center, Valdecillas Virtual Hospital, Hospital San Juan de Dios, and mobile simulation unit Hospital 12 de Octubre.

The criteria for being selected as an expert required that individuals (1) were linked to successful simulation programs running for more than three years; (2) had experience designing simulation scenarios; and (3) were engaged in clinical specialty practice.

Consensus was reached after three discussion rounds, when 73 indicators were evaluated considering their plausibility, precision, and naturality. An amount of 7 indicators were then discarded to eliminate redundancy, resulting in 66 items subject to statistical analysis. Precision and naturality prevailed as systematic variables, given that plausibility could be incorporated within precision. The definition of the systematic variables follows:

Accuracy: Qualified what could be classified as realistic conceptually or physically, compared to the clinical reality reproduced.

Naturality: Qualified what could be classified as realistic at a functional or relational level, compared to the element reproduced.

### 2.2. Quantitative Study: Sample Characteristics

The study was designed considering inputs of 4 appraisers who evaluated the same clinical scenario reproduced in 8 different recreations, considering variable combinations of the three dimensions of realism: simulated participant, scenography, and the simulator, as presented in Table 1.

Each dimension had two possible characterizations, here described:

Simulated participant could be qualified as (1) a professional actor, trained and certified by a third party, who guaranteed acting consistency between scenarios based on a role script through paid performance; or (2) an amateur actor, not trained nor certified by a third party, who voluntarily played the role of a patient with basic briefing on the clinical case and expected behavior.

Simulator could be qualified as (1) an advanced simulator/mannequin linked to software that allowed programmed responses through electronic management; or (2) a basic simulator/mannequin not linked to software nor operated through electronic management.

Scenography could be qualified based on the context the scenarios took place, which could be (1) within a Gesell Chamber—room conditioned to allow direct observation of clinical scenario without interference of spectators through mirrored glass and support of audio-video resources; or (2) with no Gesell Chamber.

The sampling was based on a completely randomized factorial design 2^3^ that resulted in the casuistry totaling 32 measurements, all scenarios featured by medicine undergraduate students as part of their simulation curricular activities.

Four appraisers conducted the quantitative assessment of the sample. Two were experts meeting the same criteria established for the Delphi study described beforehand, and two were instructors and support teaching staff, active members of a consolidated simulation program, but that were not engaged in clinical specialty practice.

Indicators were tied to a 10-point Likert scale, where one (1) was the minimum possible score for realism and ten (10) was the highest possible score. Bisquerra (2015) [19] recommends a 10-point Likert scale to allow sufficient degrees of discrimination, to have broader cutoff points to qualify the ranges, to increase the sensitivity of the instrument, and not to concur in loss of potentially discriminating data. For this study, the ranges established are:

- 1–3.5 very low
- 3.5–5 low
- 5–7 average
- 7–8.5 high
- 8.5–10 very high.

Appraisers were provided with a digital template/framework via email, instructed not to leave any field blank, and to send individual results to the research team independently for consolidation and analysis.

### 2.3. Statistical Analysis

Statistical analysis of the sample was performed using R v4.02 software (Foundation for Statistical Computing, Vienna, Austria), extracting basic quantitative indicators for the variables studied, such as the mean, median, maximum, minimum, absolute, and relative frequencies. For the statistical comparison of quantitative variables between groups, *t*-test, ANOVA, the Mann–Whitney test, and the Kruskal–Wallis test were applied, considering equality between groups as the null hypothesis. Compliance with the application criteria was evaluated using the Shapiro–Wilk normality test and the Levene test for homogeneity of variances. For qualitative variables, the existence of differences between groups was analyzed using the Chi-Square, Fischer’s exact, or Likelihood Ratio tests, considering equality between groups as the null hypothesis. Compliance with the application criteria was assessed using the Cochran criterion.

The significance level for statistical tests was set at 5% (*p* < 0.05). The internal consistency and reliability of the questionnaire was evaluated with the inter-observer agreement (Intraclass Correlation Coefficient index (ICCk2)) and the intra-observer agreement (Cronbach’s Alpha and Guttman’s Lambda 6 index (G6)).

Through the analysis process of the 66 indicators, 13 were discarded for being redundant, resulting in 53 final indicators to conduct an exploratory factorial analysis with Promax rotation, which weighted the units of realism based on the variability expressed. It allowed for the synthesizing of the contribution of each component of realism and the calculation of it by dimensions or globally, taking global realism to mean the overall verisimilitude achieved through the realism of the simulated participant, of the scenography, and of the simulator. Finally, experts have simplified the wording of indicators to facilitate reading and interpretation, seeking to reduce fatigue of the evaluator and the tendency to apply the same rating to several indicators.

To calculate global realism through ProRealSim v.1.0, the following weights apply for each dimension:

- 50% Simulated Participant—prevalent weight based on the relevance shown in the results of the study and supported by the literature that endorses the importance of the simulated participant to create atmospheres of almost absolute realism [4,20,21,22].
- 20% Simulator—less impactful weight due to the numerous reviewed studies that suggest that the simulator has the least impact on realism, due to the great variety of typologies and challenge in reproducing all aspects of the real patient. Some systematic reviews suggest that the efficacy of the simulation depends more on the training level of the students than on the realism of the simulator [22,23,24].
- 30% Scenography—remaining percentage, but with significant weight, considering the relevance facilities and material elements have when composing ambiance to facilitate immersion of those participating in the scenario [25].

## 3. Results

**Figure 1:**
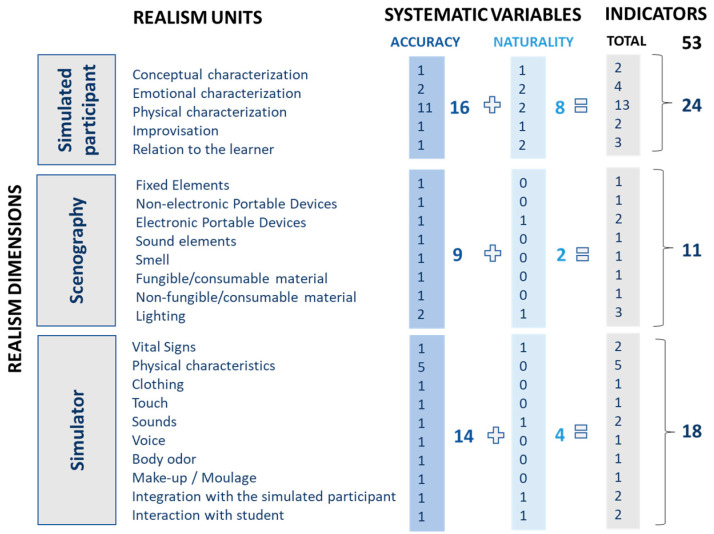
Categorization of indicators.

Figure 1 shows the categorization of the 53 indicators subject to the statistical analysis, organizing those among dimensions, units, and systematic variables.

To confirm statistical validity of the study and sample heterogeneity, no discrimination was made between experts or non-experts. Table 2 presents the means and respective ranges of appraisers’ assessment derived from the 32 data points and 53 indicators, resulting in a *p*-value significant in all cases (*p* < 0.05).

The intra-observer agreement was measured using Cronbach’s Alpha and Guttman’s Lambda 6 (G6), which provide a number between zero (0) and one (1), where one (1) denotes perfect agreement. Results are presented in Table 3 and confirm high internal consistency (≥0.969) for the assessments conducted by the same appraiser. This strengthens the reliability of the instrument within this context, with an index very close to perfect correlation, according to George and Mallery [26].

The inter-observer agreement was measured using the Intraclass Correlation Coefficient (ICC), which provides a number between zero (0) and one (1), where one (1) denotes perfect agreement. The index used was ICC2k. Table 4 presents the global agreement and agreement between evaluators. Results show that agreement and reliability was good for the simulator realism test, but poor for the simulated participant and the scenography, which showed greater dispersion.

To further analyze the inter-observer agreement, the correlation of the assessments between different observers was measured. Correlation is an indicator of the degree of linear association between two measurements that range from minus one (−1) to plus one (+1), zero (0) representing a lack of linear association.

The following figures intend to provide a visual representation of the inter-observer agreement and correlation between aspects being assessed. The ascending diagonal marks perfect correlation (+1), guiding the color code applied to the result matrix and facilitating its interpretation. The predominance of blue denotes a strong and positive correlation, versus red and white, which denote negative or inexistent correlations, respectively.

**Figure 2:**
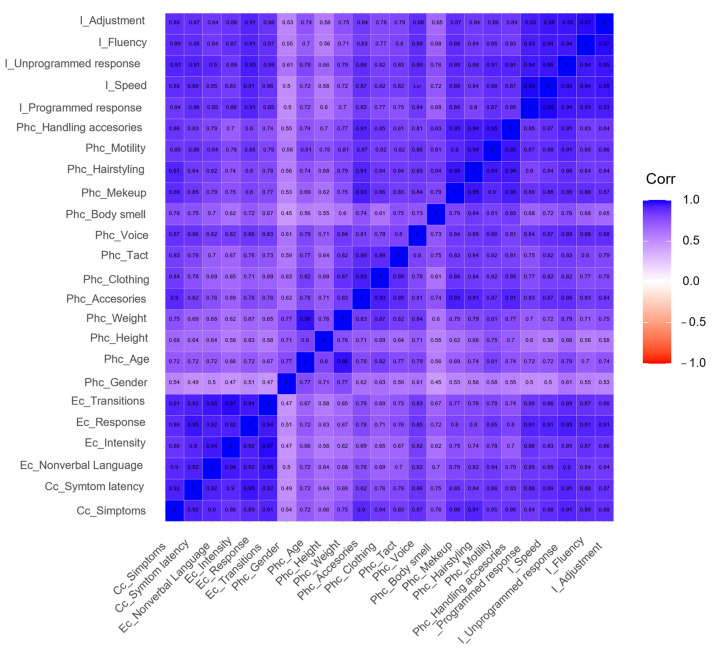
Inter-observer agreement for simulated participant. I: Improvisation, Phc: Physical characterization, Ec: Emotional characterization, Cc: Conceptual Characterization.

Figure 2 presents the correlation between the 24 indicators that characterize the dimension of Simulated Participant. A strong correlation may be observed for conceptual and emotional characterization, in addition to improvisation, confirmed by the concentration of the blue color and denoting highly consistent evaluations in those areas. Correlation for simulated participants´ phenotype indicators is more disperse, marked by light blue color lines and columns, denoting low consistency between evaluations when assessing physical characteristics.

**Figure 3:**
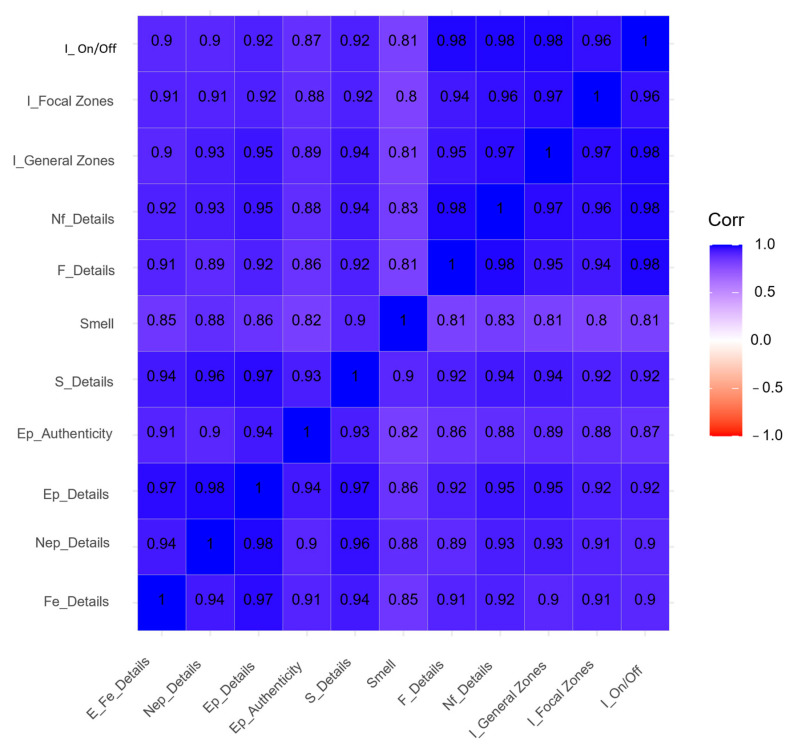
Inter-observer agreement for scenography. I: Lighting, Nf: Non-fungible/consumable material, F: Fungible/consumable material, S: Sound elements, Ep: Electronic portable devices, Nep: Non-electronic portable devices, Fe: Fixed elements.

Figure 3 presents the correlation between the 11 indicators that characterize the dimension of Scenography. A strong correlation may be observed among all indicators (all > 0.8), confirmed by how homogeneously distributed the blue color is, denoting highly consistent evaluations.

**Figure 4:**
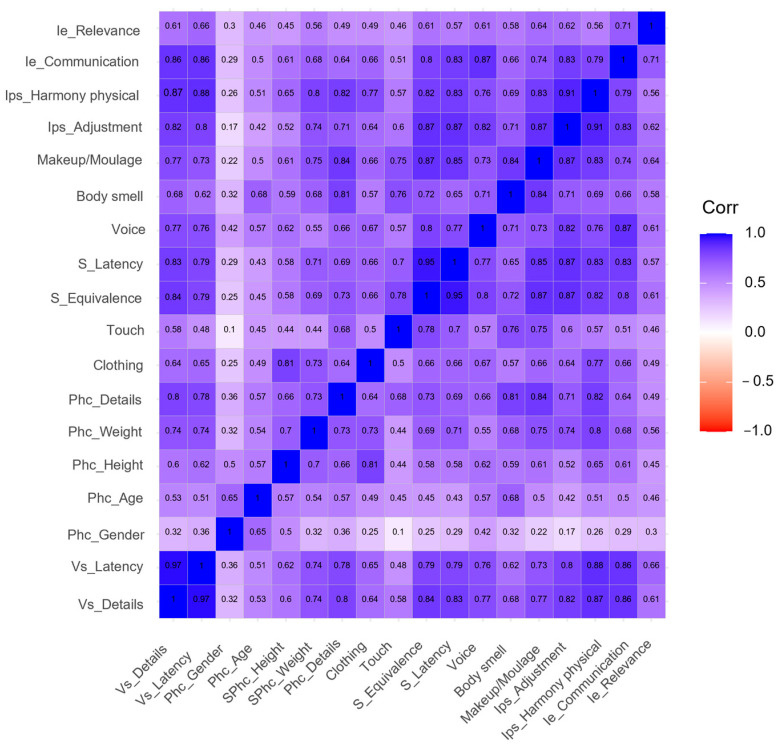
Inter-observer agreement for simulator. Ie: Interaction with the student, Ips: Integration with simulated participant, S: Sounds, Phc: Physical characteristics, Vs: Vital signs.

Figure 4 presents the correlation between the 18 indicators that characterize the dimension of Simulator. A stronger correlation may be observed by the concentration of deeper blue color for aspects related to signs sent by the simulator and level of integration with participants in the scenario, confirming consistency in evaluators’ perspective and perception. The correlation of indicators associated with simulators´ physical characteristics are more disperse, marked by white- and light blue-colored lines and columns, denoting low consistency between evaluations. This might allow us to assume that the signs given by the simulator and its interaction features with users might be more impactful on realism than its resemblance or realistic representation of phenotype. Connections between information provided by the simulator and flow of communication and dynamics of the scenario seem to be a determining factor.

To consolidate the numerical approach, an exploratory factorial analysis with Promax rotation was conducted to determine the mathematical weight to be applied to the realism units, but also to understand potential cross-impact among dimensions, as presented in Table 5.

For Simulated Participant, improvisation and relation to the learner are the most impactful factors on weighting, followed by conceptual and emotional characterization, each with similar contributions. These four units refer to the communication skills and potential of adaptability in each case, which corroborate the students´ commitment to believing and the importance of quality briefing to professional actors to integrate nuances in each scenario and maximize its effectiveness. Physical characteristics of the participant do not seem to be critical for this dimension.

For scenography, the influence of medical material, equipment, and devices seem to be key in the creation of ambiance and granting versatility to scenarios, more easily adjusting to casuistry. Most units weighted approximately the same, with the exception of smell and consumable material, which seem to have less of an impact on scenography. Nevertheless, for some clinical cases and training on diagnostic processes, smell may be a critical component, for the purpose of which additional studies might be designed.

For the simulator, all indicators related to sensorial impact on users seem to be of greatest impact in terms of mathematical weight, followed by the aspects of the simulators´ capability to relate and integrate to the user. The realistic composition of visual effects for resemblance seems to be essential, followed by sound effects.

Overall results highlight the relevance of the interaction and connection of the simulated participant with the learner as key, followed by the make-up/moulage applied on the simulator—these had the most significant individual participations within the dimensions. When considering cross-influence among dimensions and units, the simulated participant features again, considering its capacity of communicating and adapting to learners, in addition to integrating and blending in with simulators.

## 4. Discussion

After extensive review of references, no studies that evaluate the levels of global realism with objective tools [27,28] could be found. Several studies [29,30,31,32,33] show discrepancies in fidelity/realism, differing in how distinct individuals assess it (instructors and learners) and in response to variable learning objectives.

The methodology applied in this study allowed a thorough analysis of the sample and incorporated dimensions and nuances important to assessing realism, reaching statistical significance and the necessary heterogeneity for internal validation of the tool. Additionally, a structured approach was set to cluster indicators into units and dimensions.

The subjectivity inherent to all human judgment is present and may not be eliminated or isolated in the process, and the lack of culture of evaluating realism [4] should also be considered as possible biases in the study´s results. Having had included support teaching staff as appraisers might have contributed to data dispersion of the sample, given the slightly different profiles, but was also influenced by the novelty of the tool and the incipient culture of assessing realism with its intrinsic subtleness. To strengthen the criteria and data quality in future studies, relying solely on expert appraisers is recommended so that the mean (average) of all the measurements is consistent and set as a baseline.

Dieckmann et al. [34] and Reis et al. [35] mention the importance of using several expert appraisers to eliminate biases, also encouraging training to help develop the culture and experience using this type of tool. This approach would minimize ‘naive realism’ that overestimates complex technology, potentially controlling the disconnection between large economic investments in realism and the achievement of effective realism, studied by Scerbo et al. [36] and Schoenherr et al. [37].

Tied to the profile of appraisers and the data dispersion above mentioned, inter-observer agreement might be considered a fragility of the present study, but subject to further investigation. Although it had moderate rating overall, in some cases it might indicate that evaluators (expert and non-expert) score in opposite ways for the same item. This has also been observed by other authors [38] when attempting to objectify a subjective criterion, influenced by different levels of training, education, and ability.

Additionally, the intra-observer agreement in this study demonstrates strong correlations with low standard deviations. It may be interpreted as ideal, but further investigation might be conducted to eliminate possible biases of appraisers not differentiating items enough, as well as to eliminate redundant indicators. Thus, it is recommended to train appraisers in better discriminating items for a better use of the tool.

Despite these limitations, the current state of maturity of the tool allows its validation, to be followed by its open use for further development, providing more accurate results with the necessary reliability and validity. Having ProRealSim v.1.0 in digital format, bilingual, with free and open access, will favor these studies.

## 5. Conclusions

Given the methodology presented and the tested sample, the 53 indicators that compose ProRealSim v.1.0 could be considered statistically validated to offer numerical indices that objectively measure realism in clinical simulation—by dimensions and from an overall perspective. The inter- and intra-observer agreement and weighing of the dimensions and units of this study validate the indicators’ consistency, reliability, and potential to be deployed in large heterogenous samples for further refinement. By defining and categorizing the terminology related to realism among simulation professionals, negative transfer effects described by Bond in 2007 [39] due to poor realism during imperfect simulations may be avoided, reducing the negative effect in learning and error transfer to real practice.

The academic community may benefit from this robust tool to assess realism that, when appropriately used, could support the design of scenarios by promoting the ideal balance of realism necessary aligned with learning objectives. This tool provides educational benefits that, as stated by Garcovich et al., are enhanced in online access development tools [40]. It is recommended that appraisers be experienced academic, clinical, and simulation professionals, ideally trained and familiarized with the tool. At any point in time, users may contact the research and development team through the ProRealSim v.1.0 feedback tab for support, clarifications, or suggestions for improvement.

## Figures and Tables

**Table 1:** Sample distribution according to simulated scenarios.

Scenario	Simulated Participant	Simulator	Scenography
1.	Professional actor	Advanced	No Gesell chamber
2.	Amateur actor	Basic	No Gesell chamber
3.	Professional actor	Advanced	With Gesell chamber
4.	Amateur actor	Basic	With Gesell chamber
5.	Amateur actor	Advanced	With Gesell chamber
6.	Amateur actor	Advanced	No Gesell chamber
7.	Professional actor	Basic	No Gesell chamber
8.	Professional actor	Basic	With Gesell chamber

Note. Fully randomized factorial design 2^3^.

**Table 2:** Descriptive statistics of the scores assigned by the four evaluators to the eight scenarios.

	1	2	3	4	5	6	7	8	*p*	*n*
	*n* = 4	*n* = 4	*n* = 4	*n* = 4	*n* = 4	*n* = 4	*n* = 4	*n* = 4		
Simulated Participant	9.35 [9.15; 9.52]	7.83 [7.81; 7.88]	3.48 [2.56; 4.39]	6.67 [5.21; 8.29]	7.88 [7.82; 7.91]	1.83 [1.17; 2.73]	8.67 [8.26; 9.15]	8.71 [8.08; 9.24]	0.001	32
Scenography	8.55 [8.05; 9.11]	8.59 [8.45; 8.86]	1.41 [1.16; 1.66]	9.50 [9.25; 9.80]	9.41 [8.91; 9.73]	2.77 [2.43; 3.41]	8.14 [6.98; 9.16]	8.86 [8.27; 9.27]	0.004	32
Simulator	9.17 [9.01; 9.38]	7.28 [7.03; 7.43]	3.78 [3.43; 4.10]	6.75 [4.99; 8.56]	7.28 [7.25; 7.54]	3.89 [3.61; 4.19]	7.89 [6.79; 8.64]	7.72 [6.69; 8.57]	0.002	32

**Table 3:** Intra-observer agreement. Cronbach’s alpha. Guttman’s Lambda Index.

Realism Dimensions	raw_alpha	G6 (smc)
Simulated participant	0.9884208	0.9984726
Scenography	0.9915374	0.996044
Simulator	0.9698094	0.992118

**Table 4:** Agreement between evaluators. Intraclass correlation coefficient.

Realism Dimensions	ICC	F	df1	df2	*P*	Lower Bound	Upper Bound
Simulated Participant	0.3639297	3.60715	23	713	3.652659 × 10^−8^	0.2072456	0.551138
Scenography	0.1238004	2.646275	10	310	0.00413845	0.0303881	0.3348396
Simulator	0.8284964	14.45182	17	527	3.66863 × 10^−34^	0.7224845	0.9107224
Global	0.6080101	6.000236	52	1612	1.071368 × 10^−34^	0.4858323	0.7162027

Note. ICC = ICC2k. Koo and Li suggest classifying ICC values according to: <0.50 poor; 0.50–0.75 moderate; 0.75–0.90 good; >0.90 excellent.

**Table 5:** Mathematical weight of variables and their impact on dimensions.

Realism Dimensions	Realism Units	Scenography	Simulator	Simulated Participant
Simulated participant	Conceptual characterization	-	-	0.16972396
	Emotional characterization	-	-	0.14920666
	Physical characteristics	-	0.06018720	0.04449148
	Improvisation	-	-	0.20160957
	Relation to learner	-	-	0.45208840
Scenography	Fixed elements	0.16038670	-	-
	Non-electronic portable devices	0.14198533	-	-
	Electronic portable devices	0.14733311	-	-
	Sound elements	0.16541258	0.02889976	-
	Smell	0.02778539	0.03403358	-
	Fungible/consumable material	0.08664975	0.05412197	-
	Non-fungible/consumable material	0.17775232	0.06204755	-
	Lighting	0.13054525	0.06832275	-
Simulator	Vital Signs	-	0.05804105	0.03612404
	Physical characterization	0.01034069	0.06727301	-
	Clothing	0.02667548	0.04371687	-
	Touch	-	0.09380421	-
	Sounds	-	0.15384725	-
	Voice	-	0.08824702	-
	Body odor	-	0.21539492	-
	Make-up/moulage	-	0.30332866	0.01419659
	Integration with simulated participant	-	0.15610878	0.08019791
	Interaction with student	-	0.08264757	-

## Data Availability

Data will be available upon reasonable request to the corresponding author.
